# Island species radiation and karyotypic stasis in *Pachycladon *allopolyploids

**DOI:** 10.1186/1471-2148-10-367

**Published:** 2010-11-29

**Authors:** Terezie Mandáková, Peter B Heenan, Martin A Lysak

**Affiliations:** 1 Department of Functional Genomics and Proteomics, Masaryk University, and CEITEC, Masaryk University, Brno, Czech Republic; 2 Allan Herbarium, Landcare Research, Lincoln, New Zealand

## Abstract

**Background:**

*Pachycladon *(Brassicaceae, tribe Camelineae) is a monophyletic genus of ten morphologically and ecogeographically differentiated, and presumably allopolyploid species occurring in the South Island of New Zealand and in Tasmania. All *Pachycladon *species possess ten chromosome pairs (2n = 20). The feasibility of comparative chromosome painting (CCP) in crucifer species allows the origin and genome evolution in this genus to be elucidated. We focus on the origin and genome evolution of *Pachycladon *as well as on its genomic relationship to other crucifer species, particularly to the allopolyploid Australian Camelineae taxa. As species radiation on islands is usually characterized by chromosomal stasis, i.e. uniformity of chromosome numbers/ploidy levels, the role of major karyotypic reshuffling during the island adaptive and species radiation in *Pachycladon *is investigated through whole-genome CCP analysis.

**Results:**

The four analyzed *Pachycladon *species possess an identical karyotype structure. The consensual ancestral karyotype is most likely common to all *Pachycladon *species and corroborates the monophyletic origin of the genus evidenced by previous phylogenetic analyses. The ancestral *Pachycladon *karyotype (n = 10) originated through an allopolyploidization event between two genomes structurally resembling the Ancestral Crucifer Karyotype (ACK, n = 8). The primary allopolyploid (apparently with n = 16) has undergone genome reshuffling by descending dysploidy toward n = 10. Chromosome "fusions" were mediated by inversions, translocations and centromere inactivation/loss. *Pachycladon *chromosome 3 (PC3) resulted from insertional fusion, described in grasses. The allopolyploid ancestor originated in Australia, from the same or closely related ACK-like parental species as the Australian Camelineae allopolyploids. However, the two whole-genome duplication (WGD) events were independent, with the *Pachycladon *WGD being significantly younger. The long-distance dispersal of the diploidized *Pachycladon *ancestor to New Zealand was followed by the Pleistocene species radiation in alpine habitats and characterized by karyotypic stasis.

**Conclusions:**

Karyotypic stasis in *Pachycladon *suggests that the insular species radiation in this genus proceeded through homoploid divergence rather than through species-specific gross chromosomal repatterning. The ancestral *Pachycladon *genome originated in Australia through an allopolyploidization event involving two closely related parental genomes, and spread to New Zealand by a long-distance dispersal. We argue that the chromosome number decrease mediated by inter-genomic reshuffling (diploidization) could provide the *Pachycladon *allopolyploid founder with an adaptive advantage to colonize montane/alpine habitats. The ancestral *Pachycladon *karyotype remained stable during the Pleistocene adaptive radiation into ten different species.

## Background

Multiple rounds of WGD events (both allopolyploidy and autopolyploidy) cyclically increase the genetic diversity in vascular plants, and subsequently this is steadily eroded by genomic fractionation toward diploid-like genomes. Lineage-specific WGD events followed by genome repatterning and descending dysploidy toward diploid-like genomes have been revealed in several angiosperm groups and are probably best characterized in grasses [1-5] and Brassicales [6-11]. Despite still scant knowledge of the number and genealogical context of ancient WGD episodes [12], c. 15% of speciation events among extant angiosperms are associated with polyploidy [13].

Polyploidy has also played a significant role in colonization and species radiation on islands. Multiple examples of long-distance dispersals of diploid progenitors or polyploid founders followed by adaptive radiation are documented on well-studied archipelagos (Canary Islands, New Zealand, Hawaiian Islands) [14-17]. Remarkably, species radiation on islands is usually characterized by chromosomal stasis, i.e. uniformity of chromosome numbers/ploidy levels [15-17]. This means that adaptive or species radiations proceed through homoploid divergence, rather than by changing the number of linkage groups by dysploidy and/or polyploidy. The reasons for insular chromosomal stasis are most likely complex and lineage-specific, albeit the young age of radiating polyploid lines and the adaptive advantage of successful polyploid founders and their descendants are suspected as crucial factors. Although chromosomal stasis does not necessarily imply karyotypic stasis [17], only a handful of reports deal with the evolution of whole chromosome complements in island endemics. With the exception of the Hawaiian silverswords (Asteraceae), analyzed through inter-species crossing experiments and meiotic chromosome pairing configurations [18], none of the homoploid species complexes on islands has been analyzed for whole-genome collinearity.

The genus *Pachycladon *(Brassicaceae) comprises nine morphologically and ecologically diverse species in mainly alpine habitats of the South Island of New Zealand, and a single species occurs in alpine habitats in Tasmania [19,20]. The morphology of *Pachycladon *is so diverse that prior to the genus being recircumscribed by [21], species were also placed in *Cheesemania *and *Ischnocarpus*. *Pachycladon *is monophyletic [21-23], characterized by little genetic variation amongst species at a variety of genetic loci [24], and the species are interfertile [25,26]. Furthermore, six *Pachycladon *species analyzed karyologically all have the same chromosome number of 2n = 20 [27,28] and comparable genome sizes (430 to 550 Mb [26,28]). *Pachycladon *is related to *Arabidopsis *[21,23], with both these genera belonging to the polyphyletic tribe Camelineae [29,30]. The close relationship between these genera is underlined by the generation of a sexually derived intergeneric hybrid between *A. thaliana *and *P. cheesemanii *[31].

Based on chromosome counts and preliminary cytogenetic data, *Pachycladon *species were thought to have a polyploid origin (M. Lysak and P. Heenan, unpublished results). Indeed, an allopolyploid origin of the genus during the Pleistocene between ~0.8 and 1.6 mya (million years ago) has been confirmed through identification of two paralogous copies of five single copy nuclear genes [23]. Phylogenetic data of Joly et al. [23] suggested that one of the purported parents comes from the polyphyletic Camelineae or the genus *Boechera *(i.e. from crucifer lineage I [29]), whereas the *Brassica *copy from the crucifer lineage II. Our recent comparative phylogenomic study of some allopolyploid Australian Camelineae species (*Ballantinia antipoda*, 2n = 12; *Stenopetalum nutans*, 2n = 8 and *S. lineare*, 2n = 10) revealed their close phylogenetic affinity to *Pachycladon *and other Camelineae taxa [10]. The ~6 to 9 million old allopolyploid event in the ancestry of Australian genera was found to be obscured by extensive chromosome repaterrning leading to the extant diploid-like karyotypes (n = 4-6). Such concealed WGD episodes still detectable by comparative genetic and cytogenetic analysis were classified as mesopolyploid [10]. Although both recent studies [10,23] argued for an allopolyploid origin of the New Zealand and Australian Camelineae genera, the unknown genome structure of *Pachycladon *species did not yet allow to elucidate the relationship between the two polyploid Camelineae groups.

**Figure 1:**
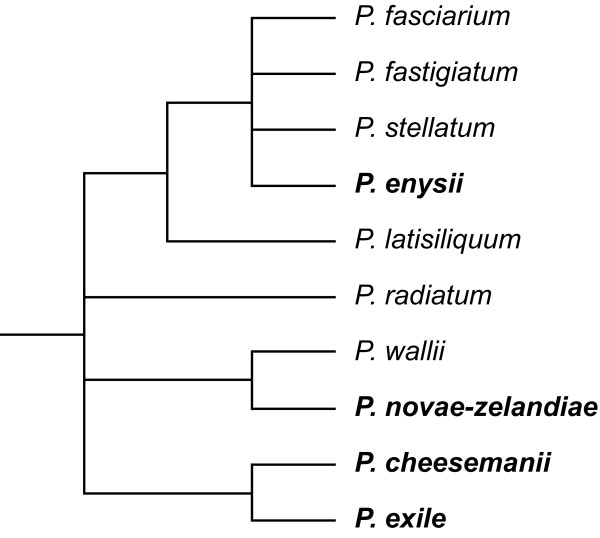
**Phylogenetic relationships in *Pachycladon***. Strict consensus tree of the six most parsimonious trees based on the internal transcribed spacer (ITS) region of 18S-25S ribosomal DNA. Species analyzed herein are in bold. Adapted from [[21], Figure 2].

In the present paper comparative chromosome painting (CCP) has been applied to four *Pachycladon *species (*Pachycladon cheesemanii*, *P. enysii*, *P. exile*, and *P. novae-zelandiae*) that represent the morphological, ecological and phylogenetic diversity of the genus (Figure 1 and [21]), and for which genetic maps are not available. *Pachycladon enysii *is a monocarpic, lanceolate and serrate-leaved, stout terminal inflorescence species of high altitude (975-2492 m) alpine greywacke rock; *P. novae-zelandiae *is a polycarpic, lobed-leaved, lateral inflorescence species of mid-altitude (1080-2031 m) alpine schist rock; and *P. chessemanii *and *P. exile *are polycarpic, heterophyllous, slender terminal inflorescence and generalist species of high fertility rock such as limestone, schist, and volcanics and occur from near sea-level to the alpine zone (10-1600 m altitude) [19]. We used CCP to study the extent of chromosome collinearity between the ten chromosomes of *Pachycladon *species and the eight chromosomes of the theoretical Ancestral Crucifer Karyotype (ACK [32,33]). Combining comparative cytogenetic data with already published accounts on phylogenetics, biogeography, and ecology of the genus we addressed (i) genome structure and evolution of *Pachycladon *species, (ii) the genome relationship to other crucifer species, particularly to the endemic Australian Camelineae taxa, and (iii) the role of major karyotypic reshuffling in the species radiation in the island setting.

## Results

### Comparative structure of *Pachycladon *karyotypes

**Figure 2:**
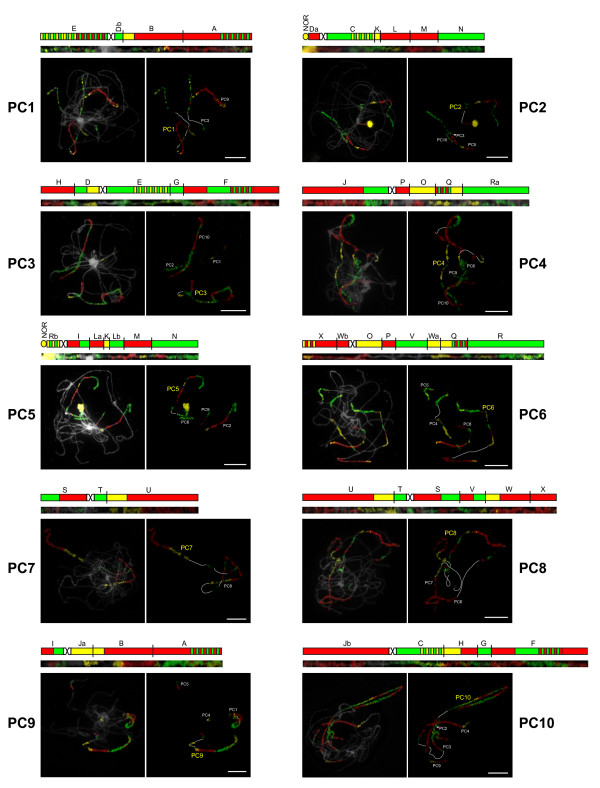
**Comparative chromosome painting (CCP) in *Pachycladon cheesemanii***. Labelling scheme, *in situ *localization within a pachytene complement and straightened pachytene bivalent for each of the ten chromosomes (PC1-PC10) are shown. Chromosomes were identified by CCP with *Arabidopsis *BAC clones and contigs labelled by biotin-dUTP (red), digoxigenin-dUTP (green), and Cy3-dUTP (yellow). Due to the duplicated nature of *Pachycladon *genomes, each painting probe labels two homeologous chromosome regions on different chromosomes (white and yellow acronyms). Chromosomes counterstained by DAPI. NOR: nucleolar organizing region. Scale, 10 μm.

**Figure 3:**
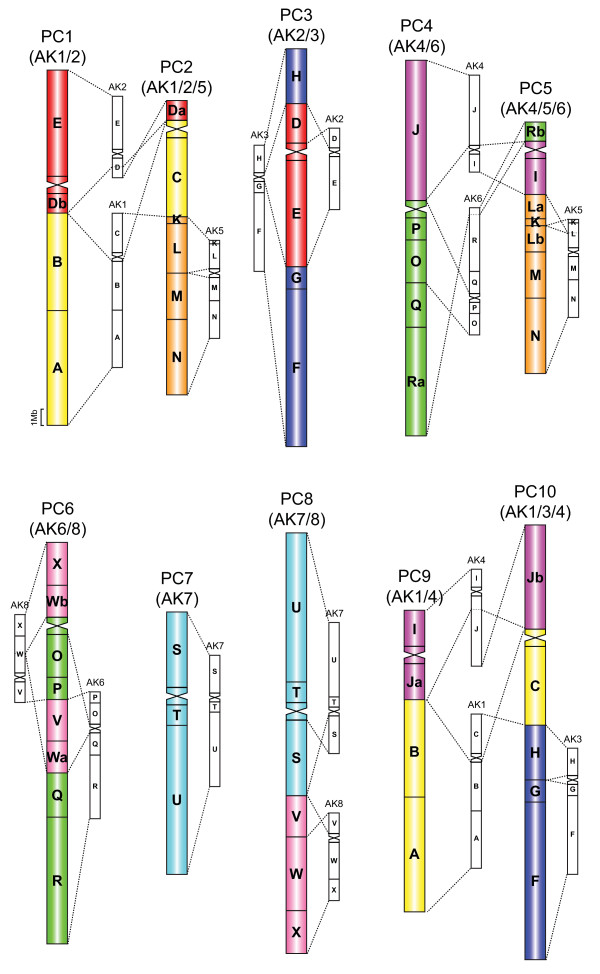
**Comparative cytogenetic map of the ancestral *Pachycladon *karyotype based on CCP data**. Collinearity relationship of the ten *Pachycladon *chromosomes (PC1 - PC10) to the duplicated Ancestral Crucifer Karyotype (ACK) comprising two sets of eight ancestral chromosomes (AK1- AK8). Dashed lines connect collinear regions shared by the two genomes. Duplicated 24 conserved genomic blocks (A-X) of the ACK are colored according to their position on chromosomes AK1 to AK8 [33]. Blocks split into two parts are labeled as „a" and „b". Centromeres of *Pachycladon *chromosomes are depicted as sandglass-like symbols colored according to their presumed origin from AK chromosomes.

The karyotype structure of four *Pachycladon *species (*P. cheesemanii*, *P. enysii*, *P. exile*, and *P. novae-zelandiae*) has been reconstructed by comparative chromosome painting (CCP) (Figure 2). Considering the close phylogenetic relationship between *Pachycladon *and *Arabidopsis *[21,23], we assumed that both genera descended from the Ancestral Crucifer Karyotype (ACK) with eight ancestral chromosomes AK1 to AK8 [32,33]. Hence *A. thaliana *BAC clones and contigs corresponding to the 24 conserved genomic blocks (GBs) of the ACK were used as painting probes to identify collinear chromosome regions in *Pachycladon *species. The four reconstructed karyotypes showed overall similarity, comprising seven (sub)metacentric (PC1, PC3, PC4, PC6 - PC8, and PC10) and three acrocentric (PC2, PC5, and PC9) chromosomes with the identical arrangement of ancestral GBs (Figure 3). The structural uniformity of all reconstructed karyotypes suggests that this structure is the ancestral *Pachycladon *karyotype.

All 24 GBs were found to be duplicated within the analyzed pachytene complements displaying regular meiotic pairing (Figure 2 and 3). The *Pachycladon *karyotype comprises one AK chromosome (PC7), seven AK-like chromosomes discernible within the composite *Pachycladon *chromosomes (four chromosomes modified by inversions), and 14 AK-like chromosome arms (Figure 3 and Table 1). Thus, in total forty-three ancestral GBs (90%) remained intact and only five blocks were split within one chromosome arm (block L on PC5), between two arms of the same chromosome (W on PC6), or between two different chromosomes (D to PC1 and PC2, J to PC9 and PC10, R to PC4 and PC5). Except chromosome PC7 resembling chromosome AK7, all *Pachycladon *chromosomes originated through "fusion" of two or three AK chromosomes (Figure 3).

**Table 1:** Comparison of ancestral genomic features between *Pachycladon* and Australian Camelineae species

	PK	BA	SL	SN
	n = 10	n = 6	n = 5	n = 4
entirely conserved AK chromosomes	**3**	4	4	3
AK chromosomes modified by inversions	**4**	2	1	4
AK chromosome arms	**14**	12	15	11
GBs not forming any AK-like structure	**2**	4	6	7
split GBs	**5**	6	6	9
lost GBs	**0**	4	2	0

non-ancestral associations of GBs	**18**	29	30	38

This table shows the extent of conservation of the eight ancestral chromosomes (AK1-8) and 24 genomic blocks (GBs) of the duplicated Ancestral Crucifer Karyotype (ACK) in *Pachycladon *and Australian Camelineae species [10]. PK- *Pachycladon *karyotype (n = 10), BA - *Ballantinia antipoda *(n = 6), SL - *Stenopetalum lineare *(n = 5), and SN - *S. nutans *(n = 4).

### Evolution of the ten *Pachycladon *chromosomes

We have reconstructed the origin of the nine "fusion" chromosomes of the ancestral *Pachycladon *karyotype using the minimal number of rearrangements and assuming that the ten PC chromosomes originated from the duplicated ACK (i.e. from 16 AK chromosomes).

#### PC1 and PC2 chromosomes

**Figure 4:**
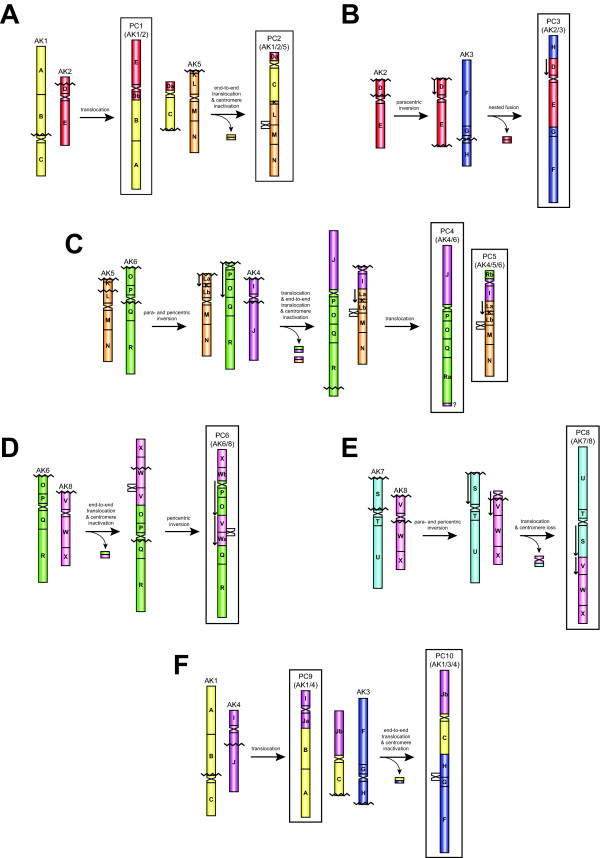
**Tentative scenarios of the origin of nine *Pachycladon *chromosomes (PC1- PC6, PC8-PC10) from the duplicated Ancestral Crucifer Karyotype (ACK)**. Duplicated 24 conserved genomic blocks (A-X) of the ACK are colored according to their position on chromosomes AK1 to AK8 [33]. Blocks split into two parts are labeled as „a" and „b". Centromeres of *Pachycladon *chromosomes are depicted as sandglass-like structures colored according to their presumed origin from AK chromosomes. Inactivated and/or lost ancestral centromeres are shown outside the modern *Pachycladon *chromosomes. Downward-pointing arrows indicate the opposite orientation of genomic blocks as compared to their position within the ACK [33]. Jagged lines mark purported breakpoints of inferred chromosome rearrangements.

(Figure 4A). PC1 originated via a reciprocal translocation between chromosomes AK1 and AK2 with breakpoints in the (peri)centromeric region of AK1 (close to block B) and in the block D of AK2. The second translocation product harbouring the AK1 centromere has been involved in a subsequent reciprocal end-to-end translocation with AK5, resulting in chromosome PC2. As the four GBs (K-N) of AK5 have the ancestral position within PC2 chromosome, we infer an inactivation and/or loss of the AK5 centromere.

#### PC3 chromosome

(Figure 4B). The origin of PC3 can be reconstructed as a paracentric inversion of the block D on AK2 followed by nested "fusion" of this chromosome into the (peri)centromere of AK3. The nested "fusion" required three or four breakpoints: two at the chromosome termini of AK2 and one or two at the centromere of AK3. One breakpoint would presumably disrupt the AK3 centromere, whereas two breaks at pericentromeric regions of the opposite arms would yield a dispensable minichromosome as a second translocation product.

#### PC4 and PC5 chromosomes

(Figure 4C). PC4 and PC5 were generated through the reshuffling of ancestral chromosomes AK4 and AK6, and AK4, AK5 and AK6, respectively. A pericentric inversion (GBs O and P) transforming AK6 into a telocentric chromosome was followed by a reciprocal translocation between this chromosome and AK4. This translocation joined the long arm of AK4 (block J) with the AK6 telocentric (= PC4). The AK4-derived telocentric chromosome comprising only the centromere and block I has undergone a reciprocal end-to-end translocation with AK5. As the GB collinearity around the AK5 centromere between blocks L and M remained conserved, we inferred an inactivation and/or loss of this centromere on PC5. A small reciprocal translocation between the bottom arm of PC4 (block R) and the upper arm of PC5 occurred after the major reshuffling steps. A paracentric inversion between GBs K and L on PC5 could have occured before the origin of both PC chromosomes or it is a later event.

#### PC6 chromosome

(Figure 4D). This chromosome most likely originated via a reciprocal end-to-end translocation between AK6 and AK8 and was probably followed by a concurrent or subsequent inactivation and/or loss of the AK8 centromere, reflected by the ancestral position of blocks V and Wa on PC6. This event was followed by a pericentric inversion with breakpoints in the (peri)centromeric region (close to block Q) and within block W.

#### PC8 chromosome

(Figure 4E). PC8 originated via a reciprocal translocation between AK7 and AK8, yielding the fusion PC8 chromosome and a meiotically unstable minichromosome containing the centromere of AK8. The translocation was preceded by a paracentric inversion on AK7 (block S) and pericentric inversion on AK8 (block V).

#### PC9 and PC10 chromosomes

(Figure 4F). Chromosome PC9 originated through a reciprocal translocation between AK1 and AK4 with breakpoints in the AK1 pericentromere (close to block B) and the proximal part of the bottom arm of AK4 (block J). The second translocation product (GBs C and Jb, and the AK1 centromere) participated in a reciprocal end-to-end translocation with AK3 which resulted in the origin of PC10 and small acentric fragment. Ancestral arrangement of AK3-derived GBs suggests that the AK3 centromere has been lost or inactivated.

The reconstructed chromosome origins are congruent with the reduction of 16 ancestral chromosomes (centromeres) to only 10 in *Pachycladon*. Centromeres of both homeologues of AK1, AK2, AK4, AK6 and AK7 remained functional, whereas six centromeres were lost (Figure 3). The centromere of one AK3 homeologue was eliminated due to the nested chromosome fusion, one AK8 centromere was eliminated via symmetric translocation, and both AK5 centromeres and centromeres of second homeologues of AK3 and AK8 were inactivated/lost (Figure 4).

Out of the 33 breakpoints inferred for the origin of ten *Pachycladon *chromosomes, 12 (36%) map to pericentromeric regions, 16 (49%) to telomeric regions, whereas only five (15%) occurred within GBs (Figure 4A to 4F).

### Chromosome landmarks (heterochromatin, telomeres and rDNA)

**Figure 5:**
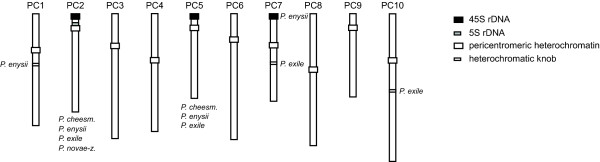
**Idiogram of ten *Pachycladon *pachytene chromosomes (PC1 to PC10) showing the positions of rDNA loci and heterochromatic arrays**. 5S and 45S rDNA loci on chromosome PC2 occur in all species analyzed; positions of other 45S rDNA loci and heterochromatic knobs are species-specific.

We have analyzed mitotic and pachytene chromosome complements of the four *Pachycladon *species for the distribution of heterochromatin domains, localization of ribosomal RNA genes (rDNA) and the *Arabidopsis*-type telomere repeat (Figure 5). Except for prominent heterochromatin of pericentromeres and terminal nucleolus organizing regions (NORs) (Figure 2), a single heterochromatic knob occurs in *P. enysii *and two knobs were found in *P. exile*. Whereas two of the three knobs reside within genomic blocks (B on PC1 in *P. enysii*, U on PC7 in *P. exile*), the knob on the bottom arm of PC10 in *P. exile *is localized between blocks G and H, i.e. at the site of presumably inactivated centromere of AK3 (Figure 4F). No heterochromatic domains were observed at the sites of other presumably inactivated and/or lost paleocentromeres. The telomere (TTTAGGG)n repeat hybridized only to chromosome ends and no interstitial telomeric signals were observed (data not shown). Whereas the four species have a single 5S rDNA locus at the same position, the number of terminal 45S rDNA loci varies. *P. novae-zelandiae *has one, *P. cheesemanii *and *P. exile *possess two, and *P. enysii *has three 45S rDNA loci, with 45S locus on the upper arm of PC2 being common to all species (Figure 5). Thus, the cross-species karyotypic stasis does not apply to the number of terminal 45S rDNA loci.

## Discussion

We have used comparative chromosome painting to reconstruct karyotype structure and evolution in the genus *Pachycladon*. Interestingly, our analysis showed that the four analyzed species representing the phylogenetic, ecological and morphological diversity of the genus possess an identical karyotype, which is also most likely to be the ancestral karyotype of the genus *Pachycladon*.

### Chromosomal and karyotypic stasis in *Pachycladon*

The present study of four *Pachycladon *species is the first whole-genome analysis of an island species radiation. *Pachycladon *species have uniformly ten chromosomes [27,28] and this infrageneric chromosomal stasis has been now extended for karyotypic stasis. Overall similar genome structures supported the monophyletic origin of the genus [21-23] and allowed inference of the ancestral *Pachycladon *karyotype whose structure remained conserved in the extant species. Karyotypic stasis revealed in *Pachycladon *clearly indicates that the Pleistocene species radiation on the South Island of New Zealand [19] was not associated with major chromosome rearrangements. The four karyotypes differ only by the number of heterochromatic knobs and NORs, without an apparent link to infrageneric phylogenetic relationships (Figure 1). Hence, the speciation proceeded through homoploid divergence from the ancestral allopolyploid genome.

Perhaps with the exception of meiotic studies in the Hawaiian silversword alliance [18] there is virtually no data on karyotype evolution during island angiosperm speciation. Hence, only the variation in chromosome number/ploidy level can be discussed more extensively. Several surveys of angiosperm chromosome numbers showed the trend of chromosomal stasis during species radiation on islands (see reviews by [15-17]). This tendency might appear paradoxical considering geographical isolation and a wealth of diverse insular environments potentially promoting the origin of novel chromosomal races and karyotypes. However, genomes diverging on islands are under multiple constraints determining chromosomal stasis or chromosomal variation. As self-evident factors influencing the insular species radiation and genomic stability are the age of islands and their distance from the mainland, the number of colonization events, the incidence of polyploidy and phylogenetic constraints. Colonizations followed by adaptive radiation on (volcanic) islands represent often relatively young evolutionary events and therefore many island endemics represent monophyletic lineages comprising closely related species with uniform chromosome numbers. Furthermore, it was concluded that chromosomal stasis vs. lability is under a strong phylogenetic constraint as some lineages (e.g. Asteraceae, *Sideritis*) seem to be more prone to genome reshuffling than others [15,16,34].

Generally the low incidence of polyploidy has been claimed for island floras [16]. These estimates collated prior to the era of indepth whole-genome analyses revealing multiple whole-genome duplications of a different age (e.g., [7,12,10]) had to be, by definition, too conservative. Recent studies suggest that colonization of islands has been frequently associated with hybridization and allopolyploidy (see [35,36] for examples). Allopolyploid ancestors originated either on continents and spread to islands (e.g. the allopolyploid ancestor of the Hawaiian mints [37]) or diploid ancestors hybridize *in situ *after long-distance dispersal (e.g. the New Zealand and Australian *Lepidium *species [38]). The allopolyploidy-driven speciation on islands is frequently associated with chromosomal stasis as shown for the Hawaiian flora with the high incidence of polyploidy (> 80% [15]).

Polyploidy is also a pronounced feature of the New Zealand flora, with 72% of the species being polyploid in families with 25 or more species [39]. Chromosomal features of New Zealand plants indicative of polyploidy are the high number of species with even haploid numbers and/or haploid numbers n = > 10-14 [40]. Many of the polyploid genera that are like *Pachycladon * exhibiting chromosomal stasis are species-rich and generally considered to be recent species radiations, often into mainly alpine-montane habitats. They include, for example, *Aciphylla *(42 species, 2n = 22), *Brachyglottis *(30 species, 2n = 60), *Chionochloa *(22 species, 2n = 42), *Gentianella *(40 species, 2n = 36), *Epilobium *(38 species, 2n = 36), and *Ourisia *(20 species, 2n = 48) (data from [27]).

Chromosomal stasis is also observed in the few crucifer genera that have species radiations on islands. All seven *Parolinia *species endemic to the Canary Islands probably have 2n = 22 (4 species counted [41]), and seven shrubby species of *Descurainia *endemic to the Canary Islands share 2n = 14 [42]. Similarly, of nine *Diplotaxis *species in the Cape Verde Islands, five have 2n = 26 [43]. Unfortunately, insufficient chromosomal data are available for c. 40 *Cardamine *species endemic to New Zealand (P. Heenan, unpublished data) as well as for most crucifer genera endemic to Australia [41,44].

### *Pachycladon *karyotype is derived from the duplicated Ancestral Crucifer Karyotype

Our data suggest that the ancestral *Pachycladon *karyotype (n = 10) was derived from the duplicated Ancestral Crucifer Karyotype (n = 8) through allopolyploidy. The ACK was expected to be inferred as an ancestral genome of *Pachycladon*, as all of the Camelineae genomes analyzed thus far have descended from the ACK (for instance, *Arabidopsis*, *Capsella*, *Turritis*, and *Neslia *[32,45,46], including the analyzed Australian Camelineae species [10]). Furthermore, the karyotypes of *Crucihimalaya *and *Transberingia*, two genera often found as being the closest relatives of *Pachycladon *[21,23], resemble the ACK structure [10]. Similarly, the ACK was proposed as an ancestral karyotype for tribes Boechereae and Cardamineae [[47], Mandáková and Lysak, unpublished data]. It is likely, therefore, that the ancestral *Pachycladon *genome has been derived from the hybridization between two ACK-like genomes. The primary allopolyploid had either the structure of duplicated ACK with n = 16 or the participating genome(s) were reduced (n = 8 → n = 7-5?) prior to the hybridization event and the allopolyploid had less than 16 chromosome pairs. The fact that paralogous genomic blocks do not lay on the same chromosome suggests that the modern *Pachycladon *chromosomes were reshuffled prior to the hybridization event, rather than due to homeologous recombination between two ACK-like genomes within the allopolyploid ancestor.

The ten composite *Pachycladon *chromosomes originated through inversions, reciprocal translocations and centromere inactivation/loss events within the duplicated ACK complement (Figure 4). Chromosome "fusions" were mediated by reciprocal translocations with or without preceding para- and pericentric inversions. These translocations yielded a "fusion" chromosome and (a)centric fragment as the second translocation product. Small acentric fragments and the minichromosome harbouring one AK8 centromere were meiotically unstable and eliminated. Whereas Robertsonian-like translocations eliminating one AK centromere together with the minichromosome is a common mechanism of the karyotype evolution in Brassicaceae [32,48,49], asymmetric translocation events yielding miniature acentric fragments and dicentric chromosomes with one AK centromere apparently inactivated or removed by recombination were proposed for the origin of composite chromosomes in the Australian Camelineae species [10]. Centromere inactivation and/or loss has been inferred on bottom (long) arms of four *Pachycladon *chromosomes (PC2, PC5, PC6, and PC10) based on the absence of ancestral centromeres and conserved organization of adjacent genomic blocks (Figure 3 and 4). The incidence of centromere inactivation in Australian and New Zealand Camelineae species might be tentatively related to the common ancestry of both lineages and/or to the duplicated character of the allopolyploid ancestral genomes. Centromere inactivation of AK4 can also be suggested for the origin of chromosome At2 in *A. thaliana *[32], and centromere inactivation of AK5 for the origin of Bst5 in *Boechera stricta *[47] and chromosome AK4/5 in *Neslia paniculata *[32]. Nevertheless, an alternative mechanism of centromere removal through subsequent paracentric and pericentric inversions followed by a symmetric translocation (Figure 2C in [32]) is also plausible, though more breakpoints have to be considered. A dicentric chromosome could also be stabilized by intrachromosomal translocation, with breakpoints in pericentromeric region of one of the ancestral centromeres, followed by a loss of the resulting centric fragment.

Chromosome PC3 originated probably through a nested "fusion" of chromosome AK2 between chromosome arms of AK3. As both AK chromosomes within PC3 possess the ancestral structure of genomic blocks (except inverted block D) translocation events with breakpoints at chromosome termini of AK2 and centromere of AK3 seems to be the parsimonious scenario. In grasses (Poaceae), insertional chromosome "fusion" has been proposed as a general mechanism of descending dysploidy [4,50], whereas in crucifers it can be suggested only for the origin of chromosome AK2/5 in *Hornungia alpina *[32]. Thus, *Pachycladon *chromosome PC3 is most likely the first instance of reconstructed insertional dysploidy in Brassicaceae. An alternative mechanism of the PC3 origin via end-to-end reciprocal translocation coupled with the elimination of the AK3 centromere requires two more breakpoints.

### Common origin of *Pachycladon *and Australian Camelineae species?

Based on the phylogenetic analysis of Australian Camelineae taxa and *Pachycladon *species, [10] concluded that both groups might originate from a very similar allopolyploid ancestor. Although the authors could not reject a single origin of both lineages, they considered two successive allopolyploidization events as more likely, i.e. mesopolyploid Australian Camelineae species originated and radiated in continental arid habitats before the mesopolyploid ancestor of *Pachycladon*. The present data corroborate this conclusion. Specifically, the two species groups do not share any cytogenetic signature, i.e. a taxon/lineage-specific chromosome rearrangement, such as the rearranged AK8 homeologue shared by five Australian species analyzed [10]. In the Australian species, any two paralogous GBs differ by the length and fluorescence intensity as revealed by CCP [10]. This difference was either present already in the hybridizing progenitors or was caused by preferential fractionation of paralogous regions belonging to only one subgenome [51]. In *Pachycladon*, two paralogous copies of all GBs cannot be distinguished upon CCP analysis. Furthermore, higher chromosome number in *Pachycladon *species (n = 10) than in the Australian species (n = 4-7) implies a more recent origin and less extensive diploidization in *Pachycladon*. Indeed, the significantly lower number of non-ancestral junctions of genomic blocks in *Pachycladon *compared to *Ballantinia antipoda *and the two *Stenopetalum *species (Table 1 and [10]) underlines the less extensive genome reshuffling in *Pachycladon*. Also the number of split GBs in *Pachycladon *(10%) is lower than in the Australian species (13% to 19%; [10]). Interestingly, both groups do not differ substantially by the number of preserved AK chromosomes, chromosome arms and GBs (Table 1). This comparison suggests that the most recent steps of chromosome number reduction in the Australian Camelineae species have been mediated by tandem end-to-end translocations followed by centromere inactivation/loss, not disrupting the structure of AK-like chromosomes and chromosome arms.

Altogether, the differences in genome structure between the mesopolyploid Australian and New Zealand lineage indicate two successive WGD events involving the same pool of parental species. The existence of the progenitor species in Australia for a long period of time is a credible assumption considering the remarkable stasis of the ACK and AK chromosomes across crucifer lineages I and II [32,33,49]. Further research is needed to elucidate if the ancient ACK-like karyotype could be found in some not yet analyzed Australian crucifer species. Recurrent formation of allopolyploids from the same or closely related parents has been documented, e.g. in the North American allopolyploid species of *Tragopogon *[52], in *Persicaria *[53] or *Arabidopsis kamchatica *[54], and also proven by the generating synthetic allopolyploids as *Arabidopsis suecica *[55], tobacco [56] or *Tragopogon mirus *and *T. miscellus *[57].

Although less likely, we cannot rule out that karyotypic change in the Australian Camelineae species and in *Pachycladon *had significantly different dynamics. The Australian Brassicaceae species exhibit a predominantly annual growth habit [44] in comparison to the perennial *Pachycladon *[21], and a more rapid rate of genome evolution could therefore be brought about with faster nucleotide substitution rates that occur in many annuals [58,59]. Perennials are thought to have greater chromosomal stasis than annuals [60,61]. Certainly the annuality could have accelerated genome reshuffling in the Australian lineage. However, for Brassicaceae we have insufficient data on large-scale genome evolution in relation to the life forms, reproduction systems and ecological factors, and as noted by [15] and [62] chromosomal evolution is often stochastic and does not obey the models.

### Phylogeographic scenario of the origin of *Pachycladon*

*Pachycladon *is the only New Zealand genus from the polyphyletic tribe Camelineae (other endemic crucifer species belong to Cardamineae, Lepidieae, and Notothlaspideae), and therefore an *in situ *origin seems unlikely. The closest Camelineae relatives of *Pachycladon *occur in Australia (e.g., *Arabidella*, *Ballantinia*, and *Stenopetalum*) and Eurasia/Beringia (e.g., *Arabidopsis*, *Crucihimalaya*, *Transberingia*) [10,23]. It seems more plausible that the hybridization event giving rise to *Pachycladon *has taken place on the Australian continent.

There are strong taxonomic and biogeographic links between Australia and New Zealand and dispersal across the Tasman Sea can occur in both directions. Tasmania and New Zealand have about 200 species in common [63], and there are many genera in continental Australia and New Zealand that have species that are closely related (e.g., *Aciphylla*, *Celmisia*, *Gentianella*, *Melicytus*, and *Ranunculus*). For these shared genera, species diversity is often highest in New Zealand and the Australian species are considered to be the result of westward dispersal from New Zealand and subsequent speciation (e.g. [64,65]). Indeed, *P. radicatum *occurs in the Tasmanian mountains and is considered to have dispersed there and diverged contemporaneously with the radiation of *Pachycladon *in New Zealand [21,22]. Other taxa are also shared between the two countries, but these are considered to have dispersed eastward from Australia to New Zealand and include, for example, *Craspedia *[66], *Montigena *[67], *Poranthera *[68], *Scleranthus *[69], and Stylidiaceae [70]. This pattern of eastward dispersal means it is plausible that *Pachycladon *could have originated in Australia and then subsequently dispersed to New Zealand.

An alternative scenario of the origin of the *Pachycladon *allopolyploid ancestor in (eastern) Eurasia followed by a later dispersal to New Zealand is unlikely and incongruent with the close phylogenetic ties of *Pachycladon *to Australian Camelineae. Also, the origin of *Pachycladon *and Australian crucifer species in New Zealand is very unlikely, considering the diversity of endemic Australian Brassicaceae taxa (15 genera and 65 species [44]).

Many of the Australian Camelineae are distributed in the arid Eremaean Zone and/or the southeastern temperate biome [44], whereas in New Zealand *Pachycladon *mainly occupies montane-alpine habitats. These three environments have expanded in both Australia and New Zealand during the Pliocene and Pleistocene and are generally considered important drivers of species radiations (e.g. [71,72]). For the Australian Camelineae their origin and diversification ~6 to 9 mya [10] is consistent with other dated molecular phylogenies of a diverse range of arid-adapted taxa [73]. These dated phylogenies show the deepest divergences of taxa are consistent with the beginning of the formation of the arid zone in the mid-Miocene and that most arid-zone species lineages date to the Pliocene or earlier. The molecular clock date of 0.8 to 1.6 mya for the origin of *Pachycladon *[23] is also consistent with its alpine distribution and habitats in the Southern Alps in the South Island of New Zealand [19]. Uplift of the Southern Alps occurred over the last 8 million years, but only reached a suitable height to permanently support alpine plants during the Pleistocene.

### Reconstructed genome evolution corroborates the close relationship of *Pachycladon *to *Arabidopsis *and other Camelineae species

The phylogenetic position of *Pachycladon *has been investigated repeatedly using various nuclear, chloroplast, and mitochondrial genes [10,21,23]. All studies are congruent in placing the genus into the crucifer lineage I, within the polyphyletic tribe Camelineae [21,29,30,74]. Although the phylogenetic relationships within Camelineae are unclear, these studies have shown *Transberingia *and *Crucihimalaya *(Camelineae), *Sphaerocardamum *(Halimolobeae), *Physaria *(Physarieae), and *Boechera *(Boechereae) to be among the closest relatives of *Pachycladon*. Based on the analysis of five single-copy nuclear genes, [23] showed that *Pachycladon *has an allopolyploid origin and that the two genomes were associated with two divergent Brassicaceae lineages (lineage I and II [29,75]). One putative parental genome was associated with Camelineae *sensu lato *(and Boechereae) and the second genome being related to Brassiceae, *Sisymbrium*, Eutremeae, Thlaspideae, and remarkably also to Cardamineae on the chalcone synthase gene tree. This pattern has been interpreted as the evidence of an inter-tribal allopolyploidization event at the origin of *Pachycladon*.

A recent study using nuclear, mitochondrial and chloroplast genes, as well as significantly increasing the sampling of Australian Camelineae (in comparison to that of [23]), has confirmed the allopolyploid origin of *Pachycladon *and provides confidence that the two gene paralogues that constitute *Pachycladon *are derived from within lineage I [10]. Most importantly, this study has disclosed the close relationship of *Pachycladon *to the Australian genera *Arabidella*, *Ballantinia*, and *Stenopetalum*, and the maternal gene paralogues of *Pachycladon *and these three genera clustered with Eurasian Camelineae (*Arabidopsis*, *Capsella, Crucihimalaya*, *Olimarabidopsis*, *Transberingia*) and North American Boechereae. The position of the paternal gene copy was less evident, but it was always embedded within lineage I, and therefore different from the study by [23]. Mandakova et al. [10] and the present study convincingly show that the *Pachycladon *ancestor orginated from hybridization between a Camelineae species and either another species of that tribe or a very closely related tribe of lineage I. Future phylogenomic analyses of other Australian crucifer genera are likely to further resolve the parentage and phylogenetic relationships of *Pachycladon*.

## Conclusion

We have shown that the remarkable infrageneric morphological and ecological differentiation in *Pachycladon *is characterized by the genome stability manifested as chromosomal and karyotypic stasis. The monophyletic *Pachycladon *species descended from a common allopolyploid ancestor (n = 10) through a whole-genome duplication of the Ancestral Crucifer Karyotype (n = 8) and subsequent diploidization by descending dysploidy. Furthermore, the present study and the phylogenetic data of [10] clearly demonstrate the close relationship between the allopolyploid *Pachycladon *and the allopolyploid Australian Camelineae taxa. CCP data demonstrate that both mesopolyploid groups most likely originated from two different WGD events that involved identical or very similar diploid parents. We argue that the *Pachycladon *ancestor has its origin in Australia and later dispersed to the South Island of New Zealand. The endemic Australian and New Zealand Camelineae provide an excellent framework to examine the nature and consequences of differently-aged WGD events within a complex of closely related species.

## Methods

### Plant material

The four species of *Pachycladon *included in this study represent the morphological and ecological diversity of the genus [19]. Plants were cultivated in a glasshouse at Landcare Research, Lincoln, New Zealand. All species have known wild origins: *P. cheesemanii *(Bobs Cove, Queenstown, Otago; 168°37'E, 47°08'S), *P. enysii *(Mount Potts, Canterbury; 170°55'E, 43°30'S), *P. exile *(Awahokomo, Otago; 170°23E, 44°42'S), and *P. novae-zelandiae *(Old Man Range, Otago; 169°12'E, 45°20'S).

### Chromosome preparation

Entire inflorescences were fixed in ethanol:acetic acid (3:1) fixative overnight and stored in 70% ethanol at -20°C until use. Selected flower buds were rinsed in distilled water and citrate buffer (10 mM sodium citrate, pH 4.8) and incubated in an enzyme mix (0.3% cellulase, cytohelicase, and pectolyase; all Sigma) in citrate buffer at 37°C for 3 h. Individual flower buds were disintegrated on a microscopic slide by a needle in a drop of citrate buffer and the suspension softened by adding 20 μL of 60% acetic acid. The suspension was spread on a hot plate at 50°C for ~0.5 min. Chromosomes were fixed by adding of ethanol:acetic acid (3:1, 100 μL) and dried with a hair dryer. Suitable slides were postfixed in 4% formaldehyde in distilled water for 10 min and air-dried.

### DNA probes for fluorescence *in situ* hybridization (FISH)

For CCP in *P. exile*, on average each third *Arabidopsis thaliana *BAC clone was used to establish contigs corresponding to the 24 genomic blocks of the ACK [33]. For the detail composition of the BAC contigs see [49]. After initial CCP experiments in *P. exile*, some BAC contigs were split into smaller subcontigs to pinpoint rearrangement of ancestral blocks. (Sub)conting characterizing chromosome rearrangements in *P. exile *were used as CCP probes to reconstruct karyotypes of *P. cheesemanii*, *P. enysii *and *P. novae-zelandiae*. The *A. thaliana *BAC clone T15P10 (AF167571) containing 45 S rRNA genes was used for *in situ *localization of NORs, and *A. thaliana *clone pCT4.2 (M65137), corresponding to a 500-bp 5 S rRNA repeat, was used for localization of 5 S rDNA loci. The *Arabidopsis*-type telomere repeat (TTTAGGG)n was prepared according to [76]. All DNA probes were labeled with biotin-dUTP, digoxigenin-dUTP, or Cy3- dUTP by nick translation as described by [49].

### FISH

To remove cytoplasm prior to FISH, the slides were treated with pepsin (0.1 mg/mL; Sigma) in 0.01 M HCl for 10 min, postfixed in 4% formaldehyde in 2× SSC (1× SSC is 0.15 M NaCl and 0.015 M sodium citrate) for 10 min, and dehydrated in an ethanol series (70, 80, and 96%). Selected BAC clones were pooled and ethanol precipitated. The pellet was resuspended in 20 μL of hybridization mix (50% formamide and 10% dextran sulfate in 2× SSC) per slide. The probe and chromosomes were denatured together on a hot plate at 80°C for 2 min and incubated in a moist chamber at 37°C overnight. Posthybridization washing was performed in 20% formamide in 2× SSC at 42°C. Detection of was as described by [49]. Chromosomes were counterstained with 4',6-diamidino-2-phenylindole (2 μg/mL) in Vectashield (Vector Laboratories). Fluorescence signals were analyzed with an Olympus BX-61 epifluorescence microscope and AxioCam CCD camera (Zeiss). Images were acquired separately for all four fluorochromes using appropriate excitation and emission filters (AHF Analysentechnik). The four monochromatic images were pseudocolored and merged using the Adobe Photoshop CS2 software (Adobe Systems). Pachytene chromosomes in Figure 2 were straightened using the plugin 'Straighten Curved Objects' [77] in ImageJ program (National Institutes of Health).

## Authors' contributions

MAL and PBH conceived the study. TM carried out the research. TM, PBH and MAL analyzed the data and wrote the manuscript. All authors read and approved the final paper.
